# Immatures of the New World treehopper tribe Amastrini (Hemiptera, Membracidae, Smiliinae) with a key to genera

**DOI:** 10.3897/zookeys.524.5951

**Published:** 2015-09-30

**Authors:** Stuart H. McKamey, Adam M. Wallner, Mitchell J. Porter

**Affiliations:** 1 Stuart H. McKamey, USDA/ARS Systematic Entomology Lab, c/o NMNH MRC-168, Smithsonian Institution, Washington, DC 20560, USA; 2 USDA-APHIS-PPQ Plant Inspection Station, P. O. Box 660520 Miami, FL 33266 USA; 3 Department of Biology, University of Maryland, College Park, MD 20742

**Keywords:** Nymph, *Amastris*, *Bajulata*, *Erosne*, *Harmonides*, *Idioderma*, *Neotynelia*, *Vanduzea*

## Abstract

The immatures stages of 8 of the 11 genera (*Amastris* Stål, *Bajulata* Ball, *Erosne* Stål, *Harmonides* Kirkaldy, *Idioderma* Van Duzee, *Neotynelia* Creão-Duarte & Sakakibara, *Tynelia* Stål, and *Vanduzea* Goding) of the tribe Amastrini are described for the first time along with brief diagnoses of Membracidae and the subfamily Smiliinae. A key to genera and notes on biology are provided. Multiple species of most genera are illustrated. Based on its distinct nymphal morphology, *Vanduzea laeta nolina* Ball is elevated to specific rank as *Vanduzea nolina* **stat. n.**, and *Bajulata*, despite the superficial similarity of its adults to those of *Vanduzea*, is confirmed as warranting generic rank based on its unique nymphal morphology. Colombia is a new country record for *Tynelia*.

## Introduction

Treehoppers (Membracidae, Aetalionidae, and Melizoderidae) are well known for the expanded, often extravagantly developed pronotum common to adults of nearly all of the more than 400 genera and 3,000 species (McKamey 1998). What is less well known is that the adult pronotum is usually displayed, in miniature form, in the last, fifth instar. In addition to this nascent enlarged pronotum, immatures are often covered with various arrangements of large spinelike structures (scoli) on the head, all thoracic segments, and the abdomen that are usually absent in the adults. Indeed, except for the nascent pronotum, treehopper immatures show a vast array of structures that to a large extent have evolved independently of the adult forms. Despite this wealth of potential diagnostic features, there has been no previous identification guide to genera of immature treehoppers.

Perhaps two of the earliest accounts of treehopper immatures were Scheller’s (1783–1794) and Fairmaire’s (1846, Pl. 3 fig. 17 of *Centrotus cornutus* [Linnaeus]). The former were cited by Buckton (1902), who also refers to “the Dutch paper read in 1868 before the Ent. Soc. of the Netherlands which treats the metamorphosis of these insects,” and reproduces images in larger form of three species. Buckton’s (1902) crude illustrations of several Neotropical species are also added, including that of a laterally compressed *Cymbomorpha* Stål nymph misidentified as *Membracis continua* Walker, which has laterally compressed adults, but not laterally compressed nymphs; Fowler (1894) also alludes to this “*Membracis*” nymph. Both references were apparently based on Westwood’s misidentification of a nymph in the Hope Collection at Oxford.

Moreover, the first accounts of any merit on New World treehopper immatures were by Funkhouser (1917), which treated and illustrated the life histories of treehoppers of the Cayuga Lake Basin in New York, and Haviland (1925), who provided brief descriptions and illustrations of the nymphs and egg masses of various species found in the Republic of Guyana. Further works are by Richter (1941a, b, 1942a, b, 1947, 1955) in Colombia; Quisenberry et al. (1978) in the United States; Strümpel (1986), who described eggs and nymphs of *Havilandia spiralis* (Haviland); Flock and Gill (1987), who illustrated and briefly described the nymph of *Parantonae* Fowler in Arizona; Pratt and Wood (1992) on the *Enchenopa binotata* (Say) species complex; Miyazaki and Buzzi (1985) on *Membracis*; Creão-Duarte and Sakakibara (1987), who described the immatures of *Kronides* Kirkaldy; Dietrich and Deitz (1991) on Aconophorini; and McKamey and Deitz (1996) on Hoplophorionini. Beyond other isolated descriptions, the features of immatures of New World taxa had received little attention until 11 characters of 56 genera (2 Amastrini genera) were included in the first phylogenetic estimate of membracid phylogeny (Dietrich et al. 2001). More recently, Godoy et al. (2006) provided images of immatures of many Neotropical genera, and Strümpel and Strümpel (2014) illustrated nymphs of a few *Enchenopa* and *Enchophyllum* species. Lencioni-Neto and Sakakibara (2013) described the immature of *Alcmeone* Stål (Darninae). The immatures of the unusual Antillean endemic genera *Antillotolania* Ramos and *Deiroderes* Ramos have also been described (McKamey and Brodbeck 2013). Regardless, formal descriptions or even illustrations of New World taxa are restricted to very few genera, and keys are nonexistant with the exception of Quisenberry et al. (1978) and Pratt and Wood (1992), severely hindering identification.

Immatures of Old world treehoppers, consisting of most of Centrotinae (Membracidae) and *Darthula* Kirkaldy (Aetalionidae) have received even less attention (Capener 1962, 1968; Ananthasubramanian 1978, 1982; Ananthasubramanian and Ghosh 1982; Ahmad and Perveen 1984, Yuan and Chou 2002). Despite having many variable features, they are morphologically less diverse than New World forms and beyond the scope of this study. No other subfamilies occur in both hemispheres.

This is the first installment describing the nymphs of New World treehopper genera, with examples of multiple species of some, egg masses of some, and other biological information. This contribution covers the tribe Amastrini, a predominantly Neotropical tribe, and includes 8 of the 11 genera (immatures of *Aurimastris* Evangelista & Sakakibara, *Hygris* Stål, and *Lallemandia* Funkhouser are unknown).

Photographs of adults of all membracid genera are available online (Deitz and Wallace 2010).

## Materials and methods

Some of the species examined are new species but their nymphal descriptions below are not intended to constitute a description recognized by the International Code of Zoological Zomenclature (International Commission on Zoological Nomenclature 1999).

Late instars of Membracidae are usually sturdy enough to maintain their form when dried, so pinned specimens were used to determine characters. The only characater that would likely be affected is the length of abdominal segment IX relative to other body parts, due to contraction. Nevertheless, this effect should be roughly equivalent in all pinned specimens.

Because some form of parental care or at least aggregation of nymphs is widespread among treehoppers, for many subfamilies it is easy to associate adults, nymphs, and eggs. In the case of solitary taxa, repeated adult-nymph-host association, rearing, and in a few cases the extrapolation of the miniature pronotom have been used to associate adults and nymphs. Generally, treehopper immatures will be identifiable to genus, and sometimes to species, so the genus is described but multiple species, if known and distinctive, are also shown. Characteristics such as oviposition style, parental care, nymphal aggregation, and ant-attendance are usually uniform within tribes. Host plants and location of feeding are also reported here as far as known.

Most vouchers are deposited in the National Museum of Natural History in Washington, D.C. (USNM) in cabinet drawers designated “McKamey et al. Membracidae Immatures Vouchers,” except those of Tynelia (see below). Each voucher also has the label “Immatures Project Voucher, McKamey et al. 2015” and the species name or, if unidentified, its species number. In most cases, additional nymphs were examined, but only those placed into the separate, synoptic collection are listed in Material examined.

Images were captured with a Microvision system and Cartograph 8.0.6 automontage software and adjusted in Adobe Photoshop. Scanning Electron Micrographs taken with A Philips XL-30 SEM using a gaseous phase on uncoated specimens.

### Morphology, characters, and terminology

The features used by (Dietrich et al. 2001) in their first phylogenetic estimate are used, except the protrusions on abdominal tergum II, which are difficult to determine in many specimens and not critical to differentiate nymphs. Also, because many states occur on the ventrolateral margins of the abdomen exist, our definition of “lamellae” has been refined, such that the nymph of *Tolania* Stål figured in the above paper (their Fig. 8b) is no longer considered as having lamellae but rather as having a row of enlarged chalazae. Dietrich et al. (2001) used 11 nymphal characters and 28 character states; and for many taxa the nymphs were unknown. Presently the current authors have 76 nymphal characters and 322 character states with multiple genera of every New World membracid tribe except Centronodini and the monobasic tribes, which are also represented. Because new characters are expected to be discovered as more higher taxa are surveyed, and it is desirable to keep characters grouped by body sections, listing the characters and character states in each paper would complicate comparative studies and use of the character matrix in future phylogenetic studies. The character descriptions and data matrix are therefore, instead, posted online on the Systematc Entomology Laboratory website (https://www.ars.usda.gov/Main/docs.htm?docid=25448). Because many of the character states have never been observed before, some of the terms used here are likewise novel or require elaboration.

**Figures 1–11.**
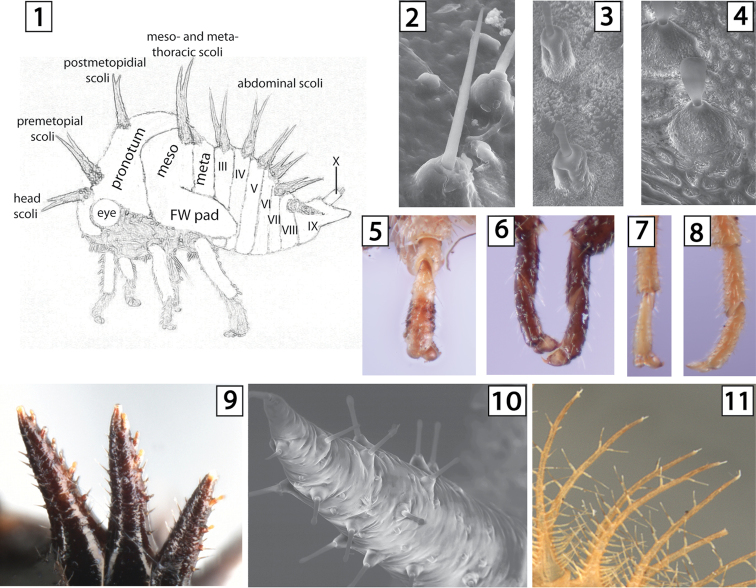
Structure and character states of Smiliinae. **1** *Neotynelia nigra*, with principal structures of an membracid nymph labelled **2–4** Needlelike, subcylindrical, and paleate setae (in *Bajulata*) of chalazae with turbuculate bases, respectively **5–6** Length of first tarsomere relative to second, distinctly shorter than, or subequal in length to, respectively **7–8** *Quadrinarea* sp., Length of pro- and mesothoracic first tarsomere relative to length of metathoracic first tarsomere **9–11** Tuberculate chalazae on scoli (**9–10**) and stalked chalazae (**11**).

The morphology of Amastrini and other Smiliinae are illustrated in Figs 1–11. As in adults, the pronotum of nymphs consists of a **premetopidium** (basally) and a **postmetopidium** (posteriorly), separated by a **metopidial sulcus**, which is usually marked by lateral callosties (see below) or by a transverse indentation. In contrast to the adults, all three of these structures provide useful characters in Smiliinae, and the pre- and postemetodidium bear scoli, or not, as if they were separate segments. **Chalazae** consist of a base, which can be tuberculate or stalked, and a seta, which can be needle- or hairlike (Fig. 2), subcylindrical and capitate (Fig. 3), or paleate (Fig. 4). Chalazae can cover the entire body, be restricted to certain areas, or be entirely absent, and enlarged chalazae can occur in various places as well, There are sometimes up to 3 **longitudinal rows** of enlarged chalazae or even scoli present on the abdomen, which sometimes extend onto the meso- and metanotum. On abdomial tergum IX, chalazae may be in paired longitudinal rows, irregularly arranged, or completely absent preapicaly and at or very near the apex. The larger, spinelike **scoli** (Figs 1, 9–11) may occur on every segment (Fig. 1), on none (Fig. 13), or some combination, often bear chalazae (Figs 9–11), and are almost always paired in the Amastrini genera currently represented and most other membracids. The placement of scoli, their basal and distal direction, and sizes relative to other scoli and lengths compared to their basal widths are all character states useful in distinguishing genera and, in some cases, species within genera. Flock and Gill (1987) illustrated scoli similar to those of Fig. 11 and referred to them as “spinose tubercles,” which is insufficient to account for the morphological variation among all membracids bearing scoli with chalazae. Instead of scoli, but in the same places, there may occur paired enlarged chalazae or paired clusters of them (Fig. 41). As noted by Dietrich et al. (2001), the enlarged chalazae (or chalazal clusters) in the same placement of scoli suggest that the enlarged chalazae are homologous. In addition to the form and arrangement of chalazae on the tibiae, an important feature of the legs is the length of the **metathoracic first tarsomere** relative to the first tarsomere of the more anterior legs (Fig. 7 vs. 8) and to the metathoracic second tarsomere (Figs 5, 6). There are also smooth **callosities** sometimes present on the head, pronotum, or mesonotum that are diagnostic.

**Figures 12–20.**
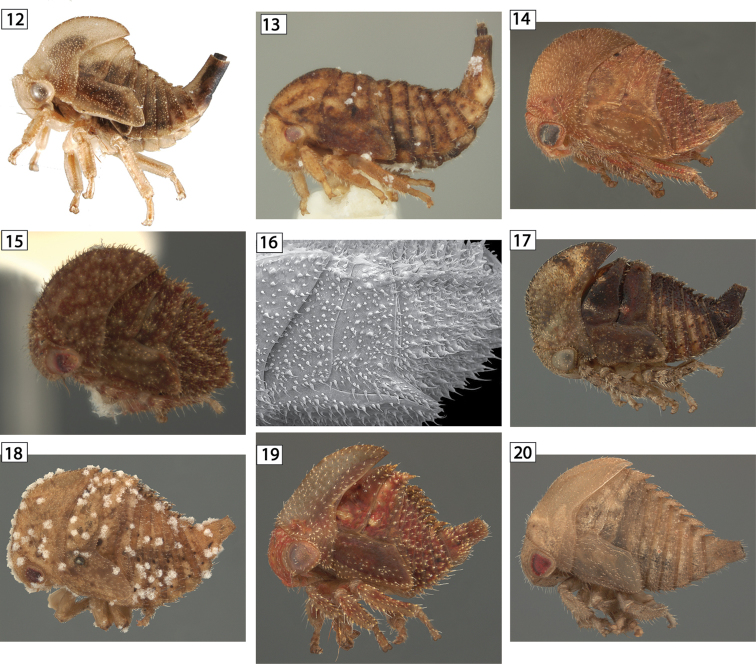
*Amastris* species in lateral view. **12** *Amastris elevata* **13** *Amastris exigua* **14** *Amastris obtegens* **15–16** *Amastris* sp. **16** habitus and detail **17** *Amastris* sp. 2. **18** *Amastris* sp. 3 **19** *Amastris* sp. 4 **20** *Amastris* sp. 5.

Characters are described from the overal body form, the head, all thoracic segments including legs and forewing wingpads, and segments III-IX of the abdomen with special emphasis on segment IX. Because new characters will undoubtedly be discovered as we explore other taxa, each of the above major regions is assigned a number and the characters are indicated by that number and a letter in the character descriptions posted online (see above). Many of these character states are related. For example, if scoli are present on the pronotum, they often occur on all thoracic and abdominal segments. There are enough exceptions, however, that separate characters are warranted for each segment. Abdominal segment X, which is the first anal segment, is sometimes long and sclerotized, but because it is often retracted into segment IX, is not included among the characters.

## Results

### Membracidae Rafinesque

Nymphs of Membracidae can be distinguished from all other Auchenorrhyncha, including the treehopper families Aetalionidae and Melizoderidae, by a ventrally fused abdominal segment IX, which thereby forms a tube through which the anal segments (X and XI) can be exerted by the nymph in defense or to proffer exudate to attendant ants, melaponine bees, or *Parachartergus* Ihering vespid wasps. Ants include the opportunistic genera *Azteca*, *Camponotus*, *Crematogaster*, and *Ectatomma*, which sometimes build vegetative enclosures around membracids, and which also collect from extrafloral nectaries and sometimes consume membracids (SHM observations, Haviland 1925). No nymphs of any of the treehopper families jump, but this is a feature shared with some leafhoppers (e.g., Eurymelinae and *Macropsis*) and some planthoppers (e.g., Tettigometridae).

Eggs of treehoppers are either laid in masses of 40–70 eggs, inserted singly or in groups of 2–5 into the tissue or not, and covered, uncovered, or enveloped by a white or brown secretion (whose characteristics have not yet been investigated) in usually distinct patterns. In Membracinae there is sometimes auxillary deposits by the female of waxlike or clear and sticky material above, below, or around the egg mass. Oviposition surfaces include crossvein leaf surfaces, leaf midribs, petioles, tendrils, twigs and thicker stems. In most genera of Hoplophorionini (Membracinae), additional incisions are made, prior to eclosion, through which the nymphs feed.

The descriptions below and in other installments are for the fifth instar, but most features apply equally to earlier instars. In younger instars some features are more pronounced, including an increased length of the scoli relative to their basal widths and to the overall body size, an increased the length of the abdominal segment IX relative to the rest of the body, and less development of the pronotum and wing pad.

### Amastrini Goding

**Note.** Deitz (1975) characterized the adults of the tribe and listed eight genera in Amastrini: *Amastris* Stål, *Bajulata* Ball, *Erosne* Stål, *Harmonides* Kirkaldy, *Idioderma* Van Duzee, *Lallemandia* Funkhouser, *Tynelia* Stål, and *Vanduzea* Goding. *Hygris* Stål was later referred to Amastrini (Sakakibara 1998) and the genera *Neotynelia* Creão-Duarte & Sakakibara (2000) and *Aurimastris* Evangelista & Sakakibara (2007) were later described. The nymphs of *Lallemandia*, *Aurimastris* and *Hygris* are unknown. Below we provide a description of the immatures of the tribe and the other genera, and their character states are posetd online (see Methods). Members of Amastrini are subsocialso, in contrast with solitary taxa such as Darninae, the association of the immatures with adults is straightforward (Fig. 65).

**Nymphal description.** ***Overall body.*** Cross-section subtriangluar; chalazae dense on thorax and abdomen, obvious throughout body (except sparse in *Harmonides reticulata* and some *Neotynelia*); no parts of body covered with waxlike substance; overall body in dorsal view elongate. ***Head.*** Dorsal or anterior rounded protuberances absent (except present in some *Neotynelia*); chalazal bases tuberculate; compound eye surface setae present (except absent in *Idioderma* and some *Neotynelia*); no enlarged chalazae between eyes; enlarged chalazae in front of ventral margin of eye absent (except present in *Amastris exigua*); enlarged chalazae adjacent to central or dorsal margin of eye absent (except present in *Harmonides* and *Vanduzea laeta*); frons not extending over central margin of eye (exception: *Bajulata bajula*). ***Prothorax.*** Dorsal and lateral suprahumeral horn buds absent; pronotal lateral margin simple (except emarginate in *Bajulata*); postmetopidium without elevation and carination that is absent in adult; metopidial sulcus not incised, continuous with adjacent surfaces above and below it; posterior extension distally narrowly convex or acute. ***Mesothorax.*** Anterior basal side of scoli, if present, without cluster of enlarged chalazae. ***Legs.*** Prothoracic tibia form simple (except foliaceus in *Bajulata*); metathoracic tarsal length subequal to pro- and mesothoracic tarsal length; all first tarsomeres distinctly shorter than second tarsomeres. ***Abdomen.*** Terga III-IV dorsally with paired apically acute scoli (except none in *Idioderma*, a single middorsal projection in *Bajulata*, and paired apically rounded or blunt scoli in *Amastris elevata*); terga V-VI dorsally with paired apically acute scoli (except paired enlarged chalazae in *Idioderma*, single middorsal projection in *Bajulata*, paired apically rounded or blunt scoli in *Amastris elevata*); tergum VII with spaired apically acute scoli dorsally (except single middorsal projection in *Bajulata*, and paired apically rounded or blunt scoli in *Amastris elevata*); tergum VIII with paired apically acute scoli dorsally (except none in *Neotynelia* sp. 1, single middorsal projection in *Bajulata*, and paired apically rounded or blunt scoli in *Amastris elevata*); lamellae absent; scoli bearing tuberculate chalazae (expect scoli absent in *Amastris exigua*). Segment IX. Distal half in cross-section usually subtriangular; preapically covered with irregularly arranged chalazae (except paired row of enlarged chalazae in some *Neotynelia*); fused portion of segment IX distal to unfused portion; unfused portion distally not bifurcate.

**Discussion.** Although each genus of Amastrini can be distinguished within the tribe, it is difficult to neatly circumscribe Amastrini. Immatures of most amastrine genera have paired, straight scoli on abdominal terga III-VIII, some also have them on the head, or thoracic nota, or the head and all thoracic nota. The genus *Bajulata* Ball is the most divergent, in having a single middorsal process on each abdominal segment, a unique condition among Smiliinae. In contrast to other Amastrini, *Bajulata* also has paleate setae on the head and thorax.

**Key to Amastrini to 5^th^ Instars**

(excluding *Aurimastris*, *Hygris*, and *Lallemandia*)

**Table:** 

1	Postmetopidium with pair of scoli (Fig. 1)	**2**
–	Postmetopidium without scoli (Figs 12–26)	**3**
2	Chalazae of body and scoli with long setae, sternum IX posteriorly projected no further ventrally than dorsally (Fig. 35)	***Tynelia***
–	Chalazae of body and scoli with very short setae, sternum IX posteriorly projected further ventrally than dorsally (Fig. 1, 30–31, 33–34)	***Neotynelia***
3	Abdominal terga III-VIII each with single middorsal scolus (Fig. 22)	***Bajulata***
–	Abdominal terga III-VIII each with paired scoli or without any scoli	**4**
4	Abdominal scoli present and larger posteriorly (Fig. 27)	***Idioderma***
–	Abdominal scoli absent or subequal in size to each other (Fig. 20)	**5**
5	Meso- and metanota each with paired scoli (Figs 24–25)	***Harmonides***
–	Meso- and metanota without scoli (Figs 26–40)	**6**
6	Meso- and metanotum each with cluster of enlarged chalazae (Fig. 40)	***Vanduzea***
–	Meso- and metanotum without enlarged chalazae (Fig. 12)	**7**
7	Pronotum extending posteriorly beyond anterior margin of metanotum	***Erosne***
–	Pronotum extending posteriorly no further than anterior margin of metanotum (Figs 12–20)	***Amastris***

**Figures 21–28.**
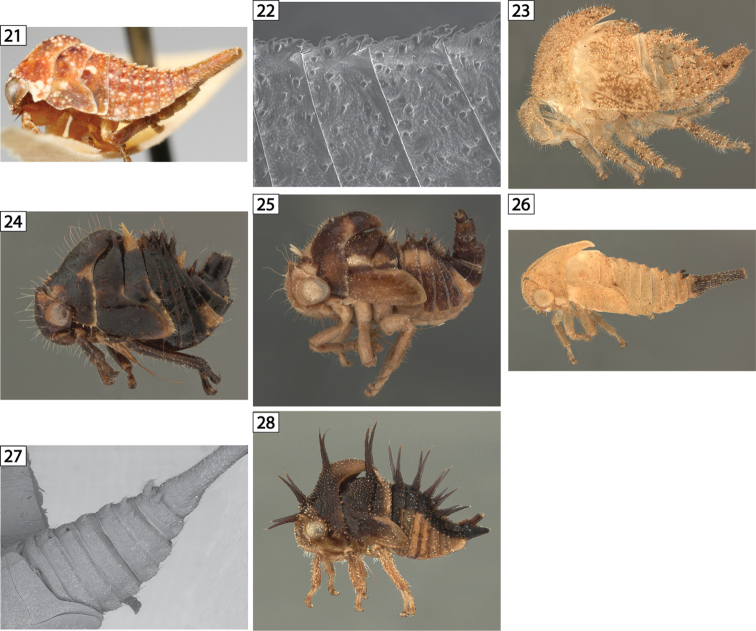
Amastrine lateral views. **21–22** *Bajulata bajula*, habitus and detail, showing the single medial scoli appressed to the following segments **23** *Erosne* sp. exuvia **24** *Harmonides reticulata* **25** *Harmonides* sp. 1. **26–27** *Idioderma virescens*, exuvia, habitus and detail **28** *Neotynelia nigra*.

**Figures 29–37.**
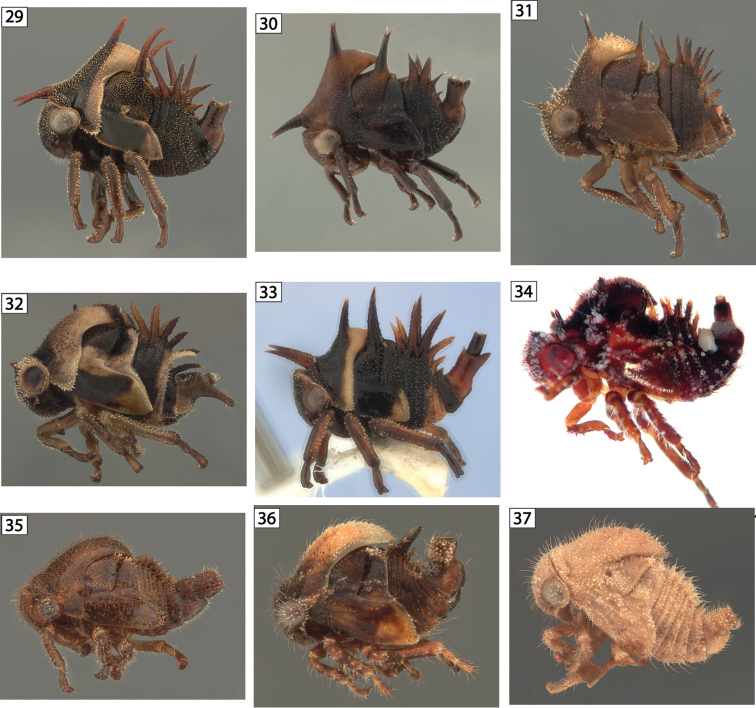
Amastrine lateral views. **29** *Neotynelia pubescens* **30** *Neotynelia* sp. 1 **31** *Neotynelia* sp. 2 (abdominal segment IX missing) **32** *Neotynelia* sp. 3 **33** *Neotynelia* sp. 4. **34** *Tynelia godoyae* (courtesy of Camilo Flóres) **35** *Vanduzea arquata* **36** *Vanduzea laeta*. **37** *Vanduzea nolina* (raised to specific rank).

**Figures 38–41.**
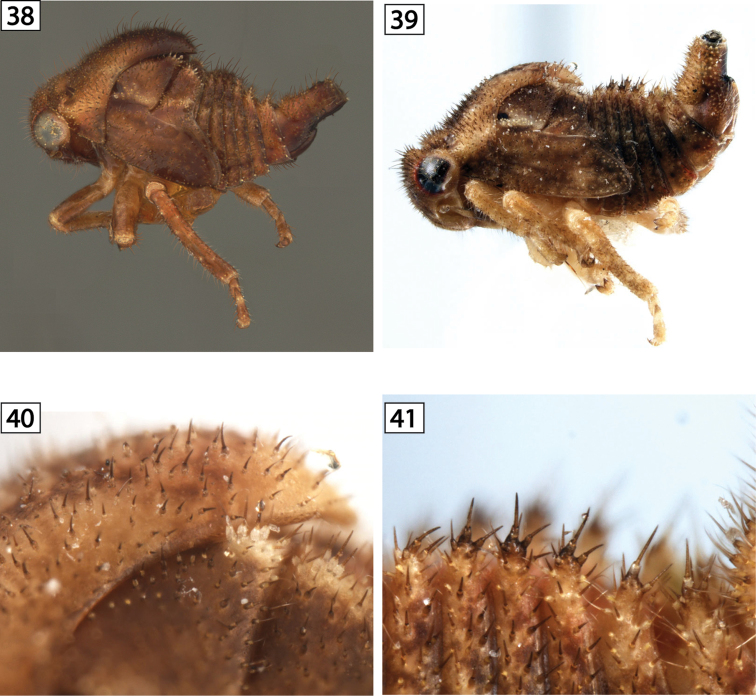
Amastrine lateral views. **38** *Vanduzea triguttata* **39–41** *Vanduzea* sp. 1 from Ecuador, habitus and detail of thoracic nota and abdominal terga.

### Amastris

Taxon classificationAnimaliaHemipteraMembracidae

Stål

Figs 12–20 , 42–49 , 65

#### Diagnosis.

Usually with the following characters: head and thorax without scoli; posterior extension of pronotum not surpassing anterior margin of metanotum; terga III-VIII with paired, short, chalazal scoli of subequal size; body including wing pad densely covered with chalazae bearing short setae.

#### Nymphal description.

***Overall body.*** Chalazal setae short; dorsal contour of abdomen in lateral view curvilinear or linear; scoli parallel (except tightly appressed in *Amastris* sp. 5). ***Head.*** Scoli absent; chalazal setae simple, needlelike. ***Prothorax.*** Pre- and postmetopidum without scoli; posterior extension of pronotum not surpassing anterior margin of metanotum. ***Mesothorax.*** With paired enlarged chalazae dorsally; scoli absent; forewing pad costal margin straight or sinuate, with enlarged chalazae only along base; forewing pad chalazae sparse, chalazal setae short; lateral rows of abdomen not extending onto meso- and metathorax. ***Metathorax.*** Dorsally with paired scoli; scolar chalazae bearing tuberculate chalazae; scolar directed dorsally or posteriorly; dorsal scoli relative size to themselves scoli 2-4 5 basal width. ***Legs.*** Chalazae of tibia on lateral margins and many on dorsal surface. ***Abdomen.*** Terga III-VIII ventrolateral margins with single enlarged chalaza; terga III-VIII dorsal scoli all subequal in size, tallest about 2-4 5 basal width, apices acute; tergum IV dorsal scoli basally directed dorsoposteriorly, distally directed posteriorly; terga III-VIII with 2 lateral rows of enlarged chalazae. Segment IX. Dorsal length subequal to combined length of segments IV-VIII; apex without dorsal enlarged chalazae or scoli; ventral extension subequal to dorsal extension.

#### Material examined.

*Amastris elevata*, 1 nymph, 1 ant, GUYANA: Demerera Co., nr Lukabuna Crk, Georgetown-Linden Hwy, ca km55, 13 July 1987, elev. ca 50m. S.H. McKamey lot #87-14c (USNM); *Amastris obtegens* (Fabricius), 1 adult, 1 nymph, Mazaruni-Potaro, ca 13 rd km S Bartica, ca 100m, 17 August 1987, S.H. McKamey lot#87-0817-g (USNM); *Amastris* sp. 1, 1 adult, 1 nymph, Mazaruni-Potaro, Bartica, 94m. 8 August 1987, S.H. McKamey lot#87-140a (USNM); *Amastris exigua* Broomfield, 1 adult, 1 nymph, ECUADOR: Pastaza-Puyo, 960m, 2 March 1986, S.H. McKamey lot#86-0302-34 (USNM); *Amastris* sp. 2, 1 adult, 1 nymph, Napo. Coca. 9–19 February 1986, 249m. McKamey, Coll. #86-0212-10 (USNM); *Amastris* sp. 3, 1 adult, 1 nymph, Moroni-Santigao. Macas, 9 May 1986, 1070m, S.H. McKamey lot# 86-0509-2 (USNM). *Amastris* sp. 4, 1 adult, 1 nymph, GUYANA, Demerara Co., ca 46 rd km S of Linden, ca. 70m, 13-Aug 1987, S.H. McKamey lot#87-160a,b USNM); *Amastris* sp. 5, 1 adult, 1 nymph, Pichincha, Tinalandia, 16 km E Sto. Domingo de los Colorados. 16–20 April 1986. ca 600m, S.H. McKamey lot#86-0419-2 (USNM); *Amastris* sp. 6, 2 adults, 1 nymph, Napo, Limoncocha, 22 August 1988, S.H. McKamey coll#88-42d (USNM).

#### Distribution.

Brazil and Peru northward to the United States.

#### Biology.

As far as known, all *Amastris* are subsocial, with the female parent sitting atop her uncovered egg mass, which are inserted into stems of the host, and tending her nymphs after hatching (Fig. 65). Nymphal aggregations are almost always ant-attended. Ant specimens pinned under vouchers include *Camponotus*, *Crematogaster*, and *Azteca*.

#### Discussion.

Broomfield (1976) revised this large genus, but many species remain undescribed, including some of the specimens examined here. Nymphs of the genus *Amastris* Stål are difficult to characterize because some species bear no distinguishing enlarged chalazae or scoli or other features and thus resemble some taxa of other Smiliinae tribes and even other subfamilies.

**Figures 42–53.**
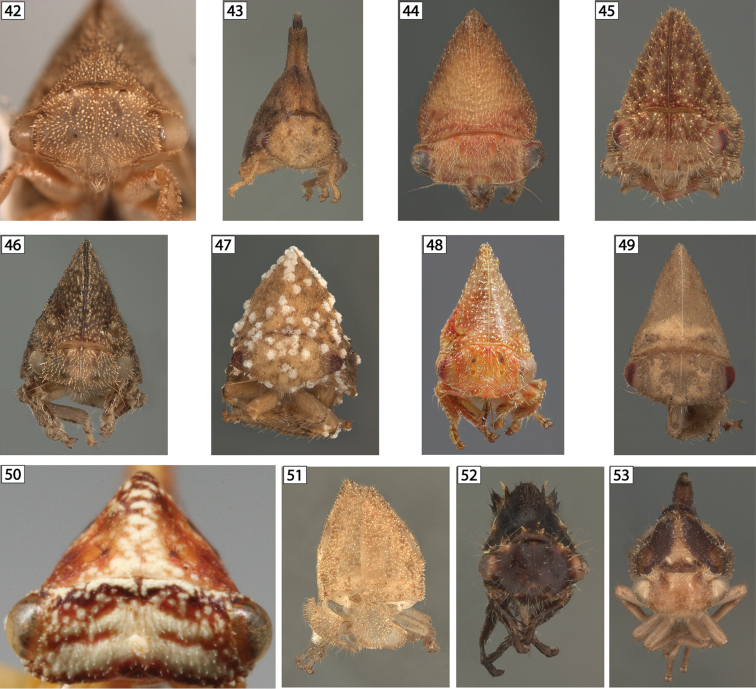
Amastrine anterior views. **42** *Amastris elevata* **43** *Amastris exigua* **44** *Amastris obtegens* **45** *Amastris* sp. 1 **46** *Amastris* sp. 2 **47** *Amastris* sp. 3 **48** *Amastris* sp. 4 **49** *Amastris* sp. 5 **50** *Bajulata bajula* **51** *Erosne* sp. exuvia **52** *Harmonides reticulata* **53** *Harmonides* sp. 1.

### Bajulata

Taxon classificationAnimaliaHemipteraMembracidae

Ball

Figs 21–22 , 50

#### Diagnosis.

head and thorax without scoli; abdominal terga each with single middorsal, heavily chalazal scolus; head and thorax with paleate setae.

#### Nymphal description.

***Overall body.*** Chalazal setae short; dorsal contour of abdomen in lateral view linear; frons extending over central margin of eye. ***Head.*** Without scoli; chalazal setae paleate. ***Prothorax.*** Pre- and postmetopidium without scoli; posterior extension of pronotum not surpassing anterior margin of metanotum. ***Mesothorax.*** Without dorsal enlarged chalazae or scoli; forewing pad costal margin emarginate; forewing pad chalazae sparse, chalazal setae short, without costal chalazae; lateral rows of abdomen not extending onto meso- and metathorax. ***Metathorax.*** Without dorsal enlarged chalazae or scoli. ***Legs.*** Prothoracic tibia foliaceus; chalazae of tibia on anterior and posterior lateral margins, absent or very few on dorsal surface. ***Abdomen.*** Terga III-VIII ventrolateral margins with row of enlarged chalazae, dorsal scoli all subequal in size, tallest dorsal scolus about 2-4 5 basal width, dorsal scoli consisting of single middorsal projection directed posteriorly, distally appressed to following tergum; tergum IV dorsal scolus distally directed posteriorly; terga III-VIII with 2 lateral rows of enlarged chalazae. Segment IX. Dorsal length subequal to combined length of segments IV-VIII; with paired enlarged chalazae apically; ventral extension subequal to dorsal extension.

#### Material examined.

*Bajulata bajula* (Goding), 1 adult, 1 nymph, USA: Tucson, Arizona, April 1942 (USNM).

#### Distribution.

United States.

### Erosne

Taxon classificationAnimaliaHemipteraMembracidae

Stål

Figs 23 , 51

#### Diagnosis.

Head and thorax without scoli; posterior extension of pronotum slightly surpassing anterior margin of metanotum; terga III-VIII with paired, short, chalazal scoli of subequal size; body including wing pad densely covered with chalazae bearing short setae.

#### Nymphal description.

***Overall body.*** Chalazal setae short; dorsal contour of abdomen in lateral view curvilinear. ***Head.*** Scoli absent; chalazal setae simple, needlelike; ***Prothorax.*** Pre- and postmetopidium without scoli; posterior extension of pronotum slightly surpasses anterior margin of metanotum. ***Mesothorax.*** With paired cluster of enlarged chalazae dorsally; scoli absent; forewing pad anterior costal margin straight; forewing pad chalazae dense, chalazal setae short; forewing pad costal chalazae only present at base. ***Metathorax.*** With paired cluster of enlarged chalazae dorsally; scoli absent. ***Legs.*** Chalazae of tibia on anterior and posterior lateral margin and dorsal surface. ***Abdomen.*** Terga III-VIII ventrolateral margins with single enlarged chalaza; dorsal scoli all subequal in size, tallest dorsal scoli about 2-4 5 basal width; tergum IV dorsal scoli basally directed dorsally or almost so, distally directed posteriorly; terga III-VIII lateral rows not manifested; tergum III with paired dorsal, apically acute scoli. Segment IX. Dorsal length subequal to combined length of segments V-VIII; dorsally without enlarged chalazae or scoli at apex; ventral extension subequal to dorsal extension.

#### Material examined.

*Erosne* sp., 2 adults, 1 exuvia, VENEZUELA: Ed. Merida Lagunillas, 17 July 1984, S.H. McKamey, Coll. (USNM).

#### Distribution.

Brazil to northern South America.

#### Discussion.

At present, the only feature found to distinguish *Erosne* nymphs from *Amastris* is the slightly further posterior extension of the pronotum in *Erosne*, which is undoudtedly coupled with the more extensive pronotum in the adults. The *Erosne* species examined is new.

### Harmonides

Taxon classificationAnimaliaHemipteraMembracidae

Kirkaldy

Figs 24–25 , 52–53

#### Diagnosis.

Head and pronotum without scoli; meso- and metanota and terga III-VIII each with short paired scoli; body evenly covered with chalazae with long setae.

#### Nymphal description.

***Overall body.*** Chalazal setae long; dorsal contour of abdomen in lateral view curvilinear; scoli splayed away from each other. ***Head.*** Scoli present or absent; scoli, if present, directed dorsad; chalazal setae simple, hairlike. ***Prothorax.*** Premetopidium scoli present or absent; premetopidium scoli, if present, directed anteriorly, and about 2-4 5 basal width; postmetopidium scoli absent; posterior extension of pronotum surpasses anterior margin of metanotum. ***Mesothorax.*** With paired scoli dorsally; scoli bearing tuberculate chalazae or without chalazae; scoli directed anteriorly or dorsally; forewing pad costal margin straight; forewing pad chalazae sparsly, their setae short; dorsal scoli 2-4 5 basal width; forewing pad without costal chalazae. ***Metathorax.*** With paired scoli dorsally, directed dorsally or almost so, 2-4 5 basal width, bearing tuberculate chalazae. ***Legs.*** Chalazae of tibia on both lateral margins but with or without chalazae on dorsal surface also. ***Abdomen.*** Terga III-VIII ventrolateral margins with single enlarged chalaza or without enlarged chalazae; terga III-VIII dorsal scoli all subequal in size or size decreasing posteriorly, tallest dorsal scoli 2-4 5 basal width; tergum IV dorsal scoli directed dorsally or almost so; terga III-VIII lateral rows not manifested; tergum III with paired apically acute scoli dorsally. Segment IX. Dorsal length subequal to combined length of segments V-VIII or VI-VIII; without dorsal enlarged chalazae or scoli at apex; ventral extension subequal to dorsal extension.

#### Material examined.

*Harmonides reticulata* (Fabricius), 2 nymphs, 1 adult, VENEZUELA: Estado Zulia, Dist. Pirija, Tocuco, 28-29 June 1984, S.H. McKamey lot#680. (USNM). Harmonides sp. 1, 1 adult, 1 nymph, PANAMA, Barro Colorado Island, Canal Zone,VII-VIII 1942, JasZetel No. 4485 (USNM).

#### Distribution.

Brazil northward to Mexico.

#### Discussion.

*Harmonides reticulata* is a common widespread species that SHM has often found in aggretations of adults and nymphs. *Harmonides* sp. 1 is curious in that it has delicate premetopidial scoli (Fig. 25) and therefore resembles *Neotynelia*. The two genera differ in the presence, in *Neotynelia*, of scoli on the head as well.

### Idioderma

Taxon classificationAnimaliaHemipteraMembracidae

Van Duzee

Figs 26–27 , 54

#### Diagnosis.

Head and thorax without scoli; terga IV-V with paired enlarged chalazae, terga VI-VIII with short paired scoli increasing in size posteriorly.

#### Nymphal description.

***Overall body.*** Chalazal setae short; dorsal contour of abdomen in lateral view linear; scoli parallel. ***Head.*** Without scoli; chalazal setae simple, needlelike. ***Prothorax.*** Pre- and postmetopidium scoli absent; posterior extension of pronotum not surpassing anterior margin of metanotum. ***Mesothorax.*** Without dorsal enlarged chalazae or scoli; forewing pad costal margin straight, without costal chalazae; forewing pad chalazae dense, chalazal setae long. ***Metathorax.*** Without dorsal enlarged chalazae or scoli. ***Legs.*** Chalazae of tibia on lateral margins and many on dorsal surface. ***Abdomen.*** Terga III-VIII ventrolateral margins with single enlarged chalaza; terga IV-VIII dorsal structures increasing in size posteriorly, tallest dorsal scoli about as tall as basal width; tergum IV dorsal scoli directed dorsoposteriorly; terga III-VIII lateral rows not manifested; tergum III without dorsal enlarged chalazae or scoli. Segment IX. Dorsal length subequal to combined length of segments IV-VIII; without dorsal enlarged chalazae or scoli at apex; ventral extension subequal to dorsal extension.

#### Material examined.

*Idioderma virescens* Van Duzee, 1 adult, 1 nymph. CUBA, Mi. 8407, VIII-3-59-18112, (USNM); 1 nymph, USA, Florida, Indian River Co., Vero Beach, 31 May 1968, J.S. Haeget, *Serenoa repens* (Bartram) [saw palmetto, Arecaceae] (USNM).

#### Distribution.

Bahamas (South Minini Island), Cuba, Jamaica, United States (Florida).

#### Biology.

Adults and nymphs of *Idioderma virescens* aggregate, feed, and develop on *Seronoa repens* (Bartram) Small (i.e. saw palmetto palm) and *Phoenix roebelenii* O’Brien (i.e. pygmy date palm) (Kopp and Tsai 1983). This species is often found tended by several species of ants (*Pseudomyrmex brunneus* Smith, *Componotus floridanus* Buckley, and *Solenopsis invicta* Buren) and may be a vector of lethal yellowing disease of palm. This is a common and widespread species occurring in the Bahamas, West Indies, and the United States (Metcalf and Brunner 1925, Metcalf 1954, Howard et al. 1981, Deitz and Wallace 2012).

**Figures 54–64.**
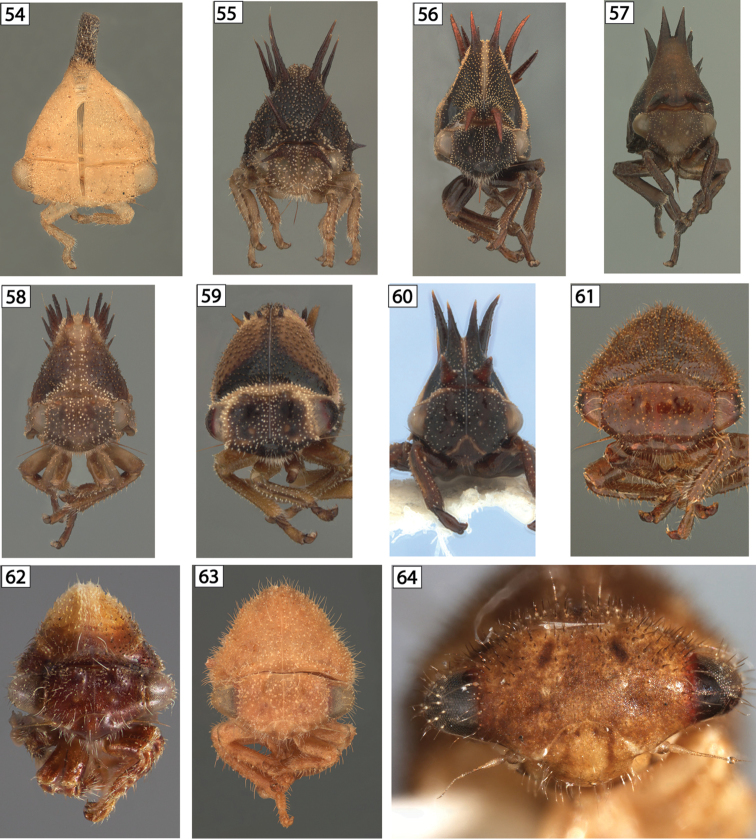
Amastrine anterior views. **54** *Idioderma virescens* exuvia **55** *Neotynelia nigra* **56** *Neotynelia pubescens* **57** *Neotynelia* sp. 1 **58** *Neotynelia* sp. 2 **59** *Neotynelia* sp. 3 **60** *Neotynelia* sp. 4 **61** *Vanduzea arquata* **62** *Vanduzea laeta* **63** *Vanduzea nolina* **64** *Vanduzea* sp. 1 from Ecuador.

**Figure 65.**
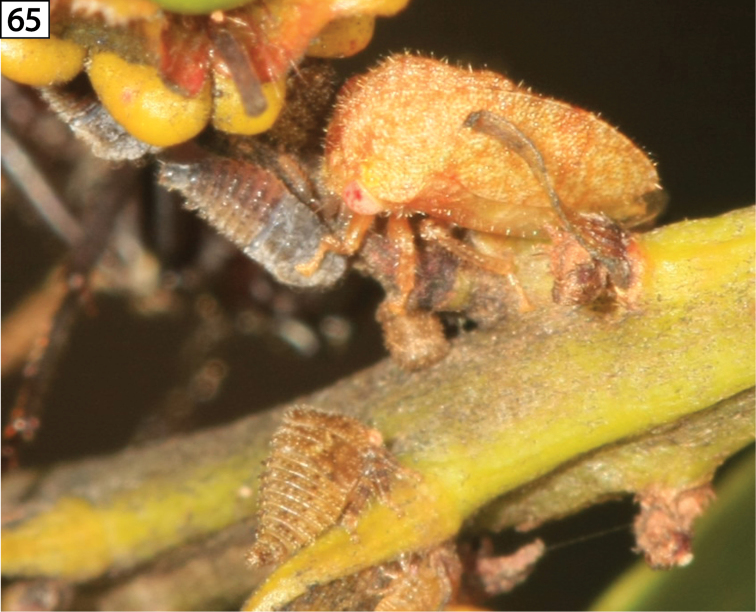
*Amastris* sp. (apparently undescribed) adult and nymphs in Brazil on *Byrsonima* sp. (Malpighiaceae), with ant in background (courtesy of Javier Ibarra Isassi).

### Neotynelia

Taxon classificationAnimaliaHemipteraMembracidae

Creão-Duarte & Sakakibara

Figs 1 , 28 , 29–33 , 55–60

#### Diagnosis.

Postmetopidium of pronotum, meso- and metanota, and abdominal terga IV-VII (usually III-VIII) each with pair of long scoli; segment IX posteriorly projected further ventrally than dorsally; body with chalazae with short subcylindrical setae; head with large or small scoli.

#### Nymphal description.

***Overall body.*** Chalazal setae short; dorsal contour of abdomen in lateral view curvilinear; scoli splayed away from each other (except scoli parallel in *Neotynelia pubescens* (Fabricius)). ***Head.*** Simple conical scoli present or absent, if present then directed anterad; chalazal setae subcylindrical. ***Prothorax.*** Premetopidium scoli present, directed anteriorly; postmetopidium scoli present, directed dorsally; posterior extension of pronotum not surpassing anterior margin of metanotum. ***Mesothorax.*** With paired dorsal scoli, directed dorsally or almost so, bearing tuberculate chalazae; forewing pad costal margin straight, without chalazae (except present on base of costal margin in *Neotynelia* sp. 4); forewing pad surface chalazae sparse with short setae; dorsal scoli usually at least 5 5 basal width, rarely 2-4 5 basal width. ***Metathorax.*** Usually with paired scoli dorsally, directed anteriorly, up to 5 5 basal width, bearing tuberculate chalazae. ***Legs.*** Chalazae of tibia on anterior and posterior lateral margins, absent or very few on dorsal surface. ***Abdomen.*** Terga III-VIII ventrolateral margins with or without single enlarged chalaza; terga IV-VII, and usually III and VIII, with dorsal scoli domen, very unequal in size, but not clinally, tallest dorsal scoli about 5 5 basal width, apically acute; tergum IV scoli basally directed dorsally or almost so, distally directed posteriorly; terga III-VIII lateral rows not manifested. Segment IX. Dorsal length subequal to combined length of segments V-VIII; apex with paired enlarged chalazae dorsally; ventral extension distinctly greater than dorsal extension.

#### Material examined.

*Neotynelia nigra* (Funkhouser), 1 adult, 2 nymphs, ECUADOR; Napo. Finca, San Jorge, ca.10 air, km E Coca, on Rio Napo. 7-10 March 1986, S.H. McKamey lot#86-0310-4, lot#86-0310-16 (USNM); *Neotynelia pubescens*, 1 adult, 1 nymph, GUYANA: Rupunini, Karanambu, 69 air, km NE Lethem, 100m. 24-26 July, 1987 S.H. McKamey lot#87-95a (USNM); *Neotynelia* sp. 1, 1 nymph (unassociated), GUYANA: Demerara, Co., Kairuni Crk., Georgetown-Linden Hwy, ca km80, ca 50m, 12 August 1987, S.H. McKamey lot#87-1746 (USNM); *Neotynelia* sp. 2, 1 nymph (unassociated) ECUADOR: Napo, Garzacocha, 68 air km E Coca. 13–17, March 1986. ca 210m, S.H. McKamey #86-0316 (USNM); *Neotynelia* sp. 3, 1 nymph (unassociated), ECUADOR: Napo 2.5 rd km E Lumbaqui, 20 January 1986. 540m, S.H. McKamey lot#86-0120-29 (USNM); *Neotynelia* sp. 4, 1 nymph (unassociated), BRAZIL: Amazonas, Parana do Xiboreninho, 03°15'-06° 00'W, 5 August 1979 (USNM).

#### Distribution.

Brazil and Peru northward to Mexico.

#### Discussion.

Contrary to the majority of *Neotynelia* species examined in this study, *Neotynelia* sp. 1 has sparse to almost absent chalazae covering its entire body.

### Tynelia

Taxon classificationAnimaliaHemipteraMembracidae

Stål

Fig. 34

#### Diagnosis.

Postmetopidium of pronotum, meso- and metanota, and abdominal terga III-VIII each with pair of long scoli; sternum IX posteriorly projected no further ventrally than dorsally; body with chalazae with hairlike long setae.

#### Nymphal description.

***Overall body.*** Chalazal setae long; dorsal contour of abdomen in lateral view curvilinear; scoli splayed away from each other. ***Head.*** Simple conical scoli present, directed anterally; chalazal setae hairlike. ***Prothorax.*** Premetopidium scoli present, directed dorsoanteriorly; postmetopidium scoli present, directed dorsally; posterior extension of pronotum not surpassing anterior margin of metanotum. ***Mesothorax.*** With paired scoli dorsally, directed dorsally or almost so, bearing tuberculate chalazae; forewing pad costal margin straight; forewing pad chalazae sparse, chalazal setae long, without costal chalazae; dorsal scoli 2-4 5 basal width; condition of lateral rows undetermined. ***Metathorax.*** With paired scoli dorsally, directed dorsoanteriorly, length 2-4 5 basal width, bearing tuberculate chalazae. ***Legs.*** Chalazae of tibia on anterior and posterior lateral margins, absent or very few on dorsal surface. ***Abdomen.*** Terga III-VIII ventrolateral marginal condition undetermined; terga III-VIII with paired dorsal scoli, unequal in size, but not clinally; terga III-VIII tallest dorsal scoli 2-4 5 basal width, apically acute; tergum IV dorsal scoli directed dorsally or almost so; terga III-VIII with lateral rows. Segment IX. Dorsal length subequal to combined length of segments V-VIII; apex with paired enlarged chalazae dorsally; ventral extension subequal to dorsal extension.

#### Material examined.

*Tynelia godoyae* Creão-Duarte & Sakakibara, images of 3 nymphs, 1 adult, COLOMBIA. Antioquia. Remedios, vereda La Cruz, finca La Brillantina, N 6.8840833 W 74.5713056, 500m, rastrojo manual, abr-2014, C. Flórez (Camilo Flórez collection).

#### Distribution.

Brazil, Peru, and Colombia.

#### Discussion.

This voucher material represents a new country record for both the genus and species. *Tynelia* most closely resembles *Neotynelia*, but differs in having long chalazal setae and in lacking the extended ventral apex of abdominal segment IX.

### Vanduzea

Taxon classificationAnimaliaHemipteraMembracidae

Goding

Figs 35–37 , 38–41 , 61–64

#### Diagnosis.

Head and thorax without scoli; meso and metanota with enlarged cluster of chalazae (Fig. 40), terga III-VIII each with pair of short, densely chalazal scoli (Fig. 41).

#### Nymphal description.

***Overall body.*** Chalazal setae long; dorsal contour of abdomen in lateral view curvilinear; scoli parallel. ***Head.*** Scoli absent; chalazal setae simple, hairlike. ***Prothorax.*** Without pre- or postmetopidium scoli; posterior extension of pronotum surpassing anterior but no posterior margin of metanotum. ***Mesothorax.*** With paired cluster of enlarged chalazae dorsally; scoli absent; forewing pad costal margin straight, without costal chalazae; forewing pad densely covered with chalazae, chalazal setae long. ***Metathorax.*** With paired cluster of enlarged chalazae dorsally. ***Legs.*** Chalazae of tibia on lateral margins and many on dorsal surface. ***Abdomen.*** Terga III-VIII ventrolateral margins without enlarged chalazae; terga III-VIII dorsal scoli all subequal in size, tallest dorsal scoli 2-4 5 basal width; tergum IV dorsal scoli directed dorsally; terga III-VIII lateral rows not manifested; tergum III with paired apically acute scoli dorsally. Segment IX. Dorsal length subequal to combined length of remaining visible abdominal terga or at least V-VIII; without dorsal enlarged chalazae or scoli at apex; ventral extension subequal to dorsal extension.

#### Material examined.

*Vanduzea arquata* Say, 1 adult, 1 nymph, USA: Maryland, Frederick County, Frederick, 2 August 1994, M.J. Rothschild, near Linden, Virginia, March 28, 1922 (USNM), 1 nymph, 1 adult, USA: North Carolina, Wake Co., Raleigh, 8 Aug 1986, S.H. McKamey lot #86-0808-2 (USNM), *Robinia pseudoacacia* L., Fabaceae; *Vanduzea laeta* Goding, 1 adult, 4 nymphs, USA: Tuscon, Arizona, 5-12-29 E.D. Ball (USNM); *Vanduzea nolina* Ball, 1 adult, 1 nymph, Nogales, E.D. Ball, Ar., 8-14-35, 7-30-37 (USNM); *Vanduzea segmentata* Fowler, 1 adult, 1 nymph, USA: Brownsville, Texas, 5-3-38, Los Angles Co., California (USNM); *Vanduzea triguttata* (Burmeister), 1 adult, 1 nymph, USA: Tucson, Arizona, June 16, 1933, P.W. Oman, (USNM). *Vanduzea* sp. 1, 1 adult, 1 nymph, ECUADOR (USNM).

#### Distribution.

Northern South America northward to Canada, Hawaii.

#### Biology.

Similar to other Amastrini genera, *Vanduzea* aggregate on their host plants and some species, for example *Vanduzea arquata* (Funkhouser, 1915), are tended by *Formica* ants (Funkhouser 1915, Fritz 1983, Crocroft 2003). These treehoppers feed on herbaceous and woody dicot hosts, such as *Albizia julibrisson*, *Citrus* sp., *Melilotus alba*, *Bidens alba*, *Eupetorium capillofolium*, *Lespedeza* sp., *Quercus* sp., and *Robinia pseudoacacia* (Kopp and Yonke 1973c, Dietrich et al. 1999). *Vanduzea* is a common and widespread genus in the Nearctic, Neotropics, West Indies, and has been introduced to the Hawaiian Islands (Deitz and Wallace 2012). SHM has observed them in Hawaii tended by ants, which are also introduced.

#### Discussion.

The nymph of *Vanduzea laeta* has unequal but not posteriorly increasing or decreasing sizes of abdominal scoli with greatly elongate scoli on terga IV and V (Fig. 36). In contrast, *Vanduzea laeta nolina* has abdominal scoli that are subequal in length and all short (Fig. 37), so the latter is here elevated to specific status.

## Supplementary Material

XML Treatment for Amastris

XML Treatment for Bajulata

XML Treatment for Erosne

XML Treatment for Harmonides

XML Treatment for Idioderma

XML Treatment for Neotynelia

XML Treatment for Tynelia

XML Treatment for Vanduzea
